# Prevalence and trend of hepatitis C virus infection among blood donors in Chinese mainland: a systematic review and meta-analysis

**DOI:** 10.1186/1471-2334-11-88

**Published:** 2011-04-09

**Authors:** Xiaofei Gao, Qian Cui, Xiang Shi, Jing Su, Zhihang Peng, Xin Chen, Na Lei, Keqin Ding, Lu Wang, Rongbin Yu, Ning Wang

**Affiliations:** 1 Department of Epidemiology and Biostatistics, School of Public Health, Nanjing Medical University, Nanjing 210029, PR China; 2 The First Clinical Medical College of Nanjing University, Nanjing 210029, PR China; 3 Department of Epidemiology, National Center for AIDS/STD Control and Prevention, Chinese Center for Disease Control and Prevention, Beijing 100050, PR China

**Keywords:** hepatitis C virus, infection, blood donors, meta-analysis

## Abstract

**Background:**

Blood transfusion is one of the most common transmission pathways of hepatitis C virus (HCV). This paper aims to provide a comprehensive and reliable tabulation of available data on the epidemiological characteristics and risk factors for HCV infection among blood donors in Chinese mainland, so as to help make prevention strategies and guide further research.

**Methods:**

A systematic review was constructed based on the computerized literature database. Infection rates and 95% confidence intervals (95% CI) were calculated using the approximate normal distribution model. Odds ratios and 95% CI were calculated by fixed or random effects models. Data manipulation and statistical analyses were performed using STATA 10.0 and ArcGIS 9.3 was used for map construction.

**Results:**

Two hundred and sixty-five studies met our inclusion criteria. The pooled prevalence of HCV infection among blood donors in Chinese mainland was 8.68% (95% CI: 8.01%-9.39%), and the epidemic was severer in North and Central China, especially in Henan and Hebei. While a significant lower rate was found in Yunnan. Notably, before 1998 the pooled prevalence of HCV infection was 12.87% (95%CI: 11.25%-14.56%) among blood donors, but decreased to 1.71% (95%CI: 1.43%-1.99%) after 1998. No significant difference was found in HCV infection rates between male and female blood donors, or among different blood type donors. The prevalence of HCV infection was found to increase with age. During 1994-1995, the prevalence rate reached the highest with a percentage of 15.78% (95%CI: 12.21%-19.75%), and showed a decreasing trend in the following years. A significant difference was found among groups with different blood donation types, Plasma donors had a relatively higher prevalence than whole blood donors of HCV infection (33.95% *vs *7.9%).

**Conclusions:**

The prevalence of HCV infection has rapidly decreased since 1998 and kept a low level in recent years, but some provinces showed relatively higher prevalence than the general population. It is urgent to make efficient measures to prevent HCV secondary transmission and control chronic progress, and the key to reduce the HCV incidence among blood donors is to encourage true voluntary blood donors, strictly implement blood donation law, and avoid cross-infection.

## Background

Chronic infection with hepatitis C virus (HCV) is a major and growing public health problem, which could easily lead to chronic liver disease, cirrhosis and even hepatocellular carcinoma [1]. The prevention and control of HCV infection showed complexity and challenge in describing geographic distribution of HCV infection, determining its associated risk factors, and evaluating cofactors that accelerate hepatitis C progression. Estimated 170 million persons are infected with HCV worldwide and more than 3.5 million new sufferers occurred annually [2]. According to the national epidemiological survey of viral hepatitis from 1992 to 1995, average anti-HCV positive rate was 3.2% in the general Chinese population, amounting to more than 30 million infected individuals [3].

The rapid global spread of HCV is believed to have occurred primarily because of efficient transmission through blood transfusion and parenteral exposures with contaminated equipment [4]. Blood donors, particularly those that rely on blood donation as a source of income, had a very high prevalence of HCV infection [5]. Recent studies have reported that the current residual risk of transfusion-transmitted HCV infection in China is about 1 in 40,000-60,000 donations, higher than that found in more developed countries [6]. With the implemention of blood donation law in 1998, many blood centers relied on other methods to motivate donors, mostly through employer-organized blood collection, but these donors may not have been true volunteers, as they may be coerced by the employer to some extent. In recent years, the true voluntary donors are gradually becoming the main source of blood donation in many blood centers in China [7]. Among paid blood donors, the HCV prevalence has reached 5.7% or higher [8]. However, among employer-organized donors and volunteer donors, the HCV prevalence was reported at lower level between 1.1-2.3%, and 0.46%, respectively [7,9].

A large amount of studies have been done in the last decade on HCV infection and its associated risk factors among blood donors. However, many of them drew incompatible or even contradictory conclusion and the utilization of these statistics are therefore limited. This paper reviews on the available studies so as to provide comprehensive and reliable epidemiological characteristics of HCV infection among blood donors in China, which is speculated to help make prevention strategies and guide further research.

## Methods

### Literature search

Literatures on the HCV prevalence among blood donors in China were acquired through searching PubMed, Embase, China National Knowledge Infrastructure (CNKI), and Wanfang Database from 1990 to 2010. In order to search and include related studies as many as possible, we used combinations of various key words, including hepatitis C virus or HCV, blood donors, and China or Chinese Mainland.

### Selection and data abstraction

All the potentially relevant papers were reviewed independently by two investigators through assessing the eligibility of each article and abstracting data with standardized data-abstraction forms. Disagreements were resolved through discussion. The following information, though some studies did not contain all of them, were extracted from the literatures: first author's name, publication date, study period, province of sample, blood donor recruitment methods (paid blood donors, employer-organized donors, or true volunteer donors), type of blood donation (categorized as plasma donors and whole blood donors), sampling size, the number of subjects infected with HCV, HBV, and HIV or co-infected with two or three of these viruses, gender, age (18-30 years and 31-60 years), and blood type, etc.

The inclusion criteria were: (1) studies in the mentioned four databases with full text, despite the language of original text; (2) studies reporting anti-HCV positive rates among blood donors in Chinese Mainland; (3) studies using anti-HCV as a detection index of HCV. The exclusion criteria were: (1) studies without specific sample origins; (2) studies with overlapping time intervals of sample collection from the same origin; (3) studies with a sample size less than 50; (4) studies that failed to present data clearly enough or with obviously paradoxical data.

### Statistical analysis

In our review, random effect models were used for meta-analysis, considering the possibility of significant heterogeneity between studies which was tested with the Q test (*P *< 0.10 was considered indicative of statistically significant heterogeneity) and the *I^2^*statistic (values of 25%, 50% and 75% are considered to represent low, medium and high heterogeneity respectively). Freeman-Tukey arcsin transform to stabilize variances, and after the meta-analysis, investigators can transform the summary estimate and the CI boundaries back to proportions using sin function, the specific conversion details can be seen in reference [10]. Stratified analyses were performed by study locations, gender, age, study period, blood donor recruitment methods, type of blood donation, and blood type. The Z or χ^2 ^test was used to assess the differences among the subgroups. Data manipulation and statistical analyses were undertaken using the Statistical Software Package (STATA) 10.0 (STATA Corporation, College Station, TX, USA, 2009), and ArcGIS 9.3 (ESRI, Redlands, California, USA) was used for map construction.

## Results

**Figure 1:**
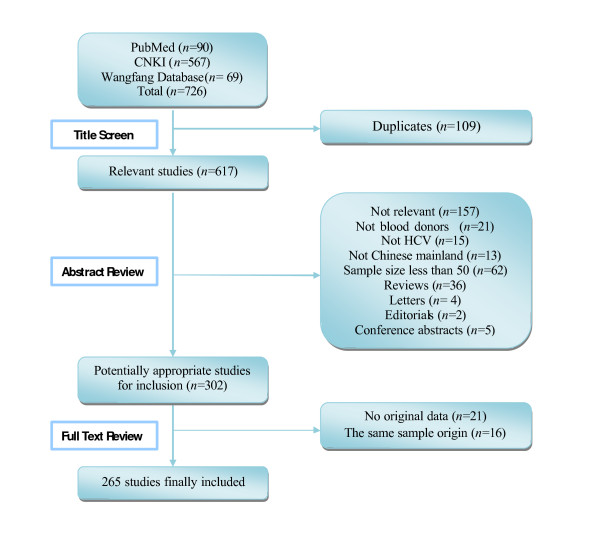
**Results of the systematic literature search**.

According to the literature search strategies, 726 studies (90 studies in PubMed, 636 studies in CNKI and Wanfang database) were identified, but 461 studies were excluded based on the inclusion and exclusion criteria (Figure 1). There were 11 studies in English [8,9,11-19] and 254 studies in Chinese [20-273] of the finally adopted 265 studies.

### General information of samples

A total of 4519313 blood donors between the ages of 18 to 60 were included, with a wide range of blood donation frequency from 1 to more than 50 times. Some donors had a duration (the period from the first blood donation to when selected in original research) longer than 15 years. The majority of blood donors were men, approximately 57.72% (101319/175540), while women accounted for 42.28% (74221/175540). The occupation of blood donors was widely distributed. Voluntary blood donors mainly came from college students, health care providers, officials, and military from Chinese People's Liberation Army (PLA), while paid blood donors were mostly from peasants, low-wage workers, and unemployed individuals.

The blood samples mainly came from blood banks, hospitals, and Centers for Disease Control and Prevention (CDC). The studies of our review involved in the following regions of 29 provinces and cities: Central China (Hunan, Hubei, Henan), North China (Beijing, Hebei, Shanxi, Tianjin, Inner Mongolia), South China(Guangdong, Guangxi), Northwest China(Shanxi, Gansu, Ningxia, Qinghai, Xinjiang), Northeast China (Liaoning, Jilin, Heilongjiang), Southwest China (Yunnan, Guizhou, Sichuan, Chongqing), East China (Shandong, Jiangsu, Anhui, Zhejiang, Fujian, Shanghai, Jiangxi).

### Prevalence of HCV infection among blood donors in Chinese mainland

#### Region

**Figure 2:**
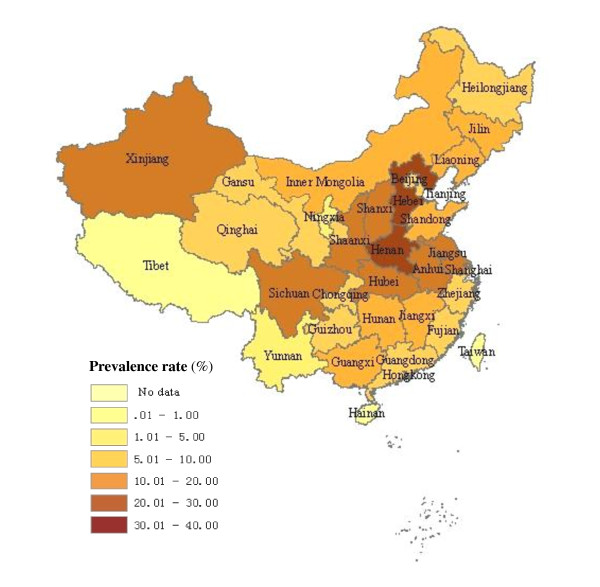
**The regional distribution of pooled prevalence of HCV infection among blood donors in China before 1998**.

**Figure 3:**
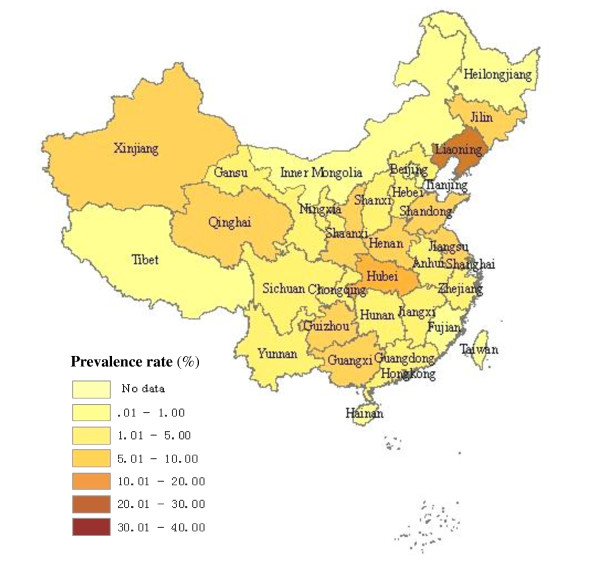
**The regional distribution of pooled prevalence of HCV infection among blood donors in China after 1998**.

As seen in Table 1 and Figure 2, 3, the pooled prevalence of HCV infection among blood donors in Chinese mainland from 1990 to 2010 was 8.68% (95% CI: 8.01%- 9.39%). Dramatic geographic difference in pooled HCV infection rates among blood donors was observed. The epidemic was severest in North and Central China, where the HCV infection rate were 13.45% (95%CI: 11.41%-15.67%) and 14.74% (95%CI: 11.06%-18.80%), respectively. The lowest prevalence was in South China with the rate of 2.88% (95% CI: 2.19%-3.64%). Before 1998, the pooled prevalence of HCV infection was 12.87% (95% CI: 11.25%-14.56%) among blood donors, with the highest rates found in Henan (35.04%, 95% CI: 23.62%-47.41%), then Hebei (29.26%, 95% CI: 19.63%-39.98%), and then the pooled prevalence decreased to 1.71% (95%CI: 1.43%-1.99%) after 1998.

**Table 1:** Prevalence of HCV infection among blood donors at different regions

Study location	Province of study	Total^b^		Before 1998		After 1998	
		
		No	Prevalence % (95%CI)	No	Prevalence % (95%CI)	No	Prevalence % (95%CI)
East China	Anhui	21	11.25(6.56,17.04)	19	12.91(8.31,18.34)	2	0.90(0.01,3.14)
	Fujian	4	3.38(2.19, 4.83)	4	3.38(2.19, 4.83)		
	Jiangsu	21	11.41(6.12, 18.07)	13	14.84(8.73,22.23)	7	3.83(0.75,9.13)
	Jiangxi	6	5.65(1.52, 12.21)	4	5.15(2.31, 9.07)	1	0.38(0.26,0.52)
	Shandong	27	6.34(5.00, 7.80)	17	8.37(5.84, 11.31)	7	1.64(1.26,2.07)
	Shanghai	4	2.59(0.38, 6.66)	4	2.59(0.38, 6.66)		
	Zhejiang	12	2.02(1.06, 3.28)	8	3.08(1.40, 5.42)	4	0.79(0.30,1.51)
North China	Beijing	9	2.95(1.78, 4.41)	7	3.53(1.84, 5.74)		
	Hebei	19	26.92(15.20,40.57)	18	29.26(19.63,39.98)	1	0.41(0.39,0.43)
	Shanxi	9	14.63(8.73, 21.74)	5	24.17(18.14,30.77)	2	0.41(0.06,2.31)
	Tianjin	5	2.35(0.98, 4.33)	2	1.97(0.32, 5.02)	2	
	IM^a^	4	9.36(3.00, 18.76)	4	9.36(3.00, 18.76)		
South China	Guangdong	13	1.05(0.65, 1.54)	5	1.41(0.87, 2.07)	8	0.91(0.49,1.47)
	Guangxi	8	9.75(6.94, 12.98)	5	17.49(1.71, 44.67)	2	3.31(0.08,10.90)
Central China	Hubei	11	10.81(5.51, 17.61)	8	13.04(6.41, 21.61)	3	5.54(0.63,14.84)
	Hunan	13	8.01(3.22, 14.70)	8	17.11(9.81, 25.95)	3	0.55(0.35, 0.81)
	Henan	26	20.63(12.98,29.53)	16	35.04(23.62,47.41)	9	3.83(1.75, 6.66)
Northwest	Shanxi	6	11.25(4.81, 19.91)	4	17.42(3.60, 38.61)	1	1.99(1.95, 2.03)
	Ningxia	2	0.69(0.10, 1.78)	1	1.18(1.03, 1.35)	1	0.32(0.29, 0.37)
	Qinghai	3	2.60(0.10, 8.34)	2	3.59(0.00, 13.69)	1	1.10(0.511.91)
	Gansu	6	2.60(5.44, 17.49)	3	23.32(3.62, 53.16)	2	0.83(0.29, 1.64)
	Xinjiang	5	14.52(3.01, 32.54)	4	19.67(3.79, 43.83)	1	1.45(1.31, 1.58)
Southwest	Sichuan	10	11.31(3.19, 23.49)	8	15.60(7.61, 25.77)	2	0.71(0.28, 1.34)
	Yunnan	1	0.23(0.17, 0.29)			1	0.23(0.17, 0.29)
	Guizhou	9	5.00(1.76, 9.78)	6	6.91(2.88, 12.51)	3	1.71(0.43, 3.81)
	Chongqing	5	1.33(0.50, 2.54)	2	2.22(0.19, 6.36)	3	0.97(0.18, 2.37)
Northeast	Liaoning	6	5.84(3.87, 8.18)	5	5.00(3.29, 7.06)	1	11.82(8.29,15.89)
	Heilongjiang	4	4.06(2.86, 5.47)	4	4.06(2.86, 5.47)		
	Jilin	5	5.38(2.26, 9.69)	3	7.19(1.08, 18.03)	2	3.59(0.17, 16.81)
Pooled		268	8.68(8.01,9.39)	189	12.87(11.25,14.56)	69	1.71(1.43, 1.99)

a. IM: Inner Mongolia

b. When calculating the pooled prevalence rate either before or after 1998 respectively, the studies that spanned 1998 were excluded. In that case, number of total literatures is bigger than the sum of literature number before 1998 and after 1998.

#### Gender

A total of 79 studies have investigated the association between the prevalence rate of HCV infection and gender among blood donors. HCV infection rate of male blood donors was 9.87% (95%CI: 8.26%-11.63%), while female blood donors had a rate of 9.78% (95%CI: 7.88%-11.89%). There was no significant statistical difference between males and females (Z = 0.62, *P *= 0.54).

#### Age

There were 30 studies indicating the association between the prevalence rate of HCV infection and age among blood donors. In most studies blood donors were divided into two groups by the age of 30 years old. HCV infection rate of individuals aged 18-30 was 4.91% (95% CI: 3.89%-6.05%), while the prevalence rate of individuals aged 31-60 was 8.99% (95% CI: 6.86%-11.37%), and there was significant statistical difference between two groups (Z = 66.02, *P *< 0.01).

#### Time

**Figure 4:**
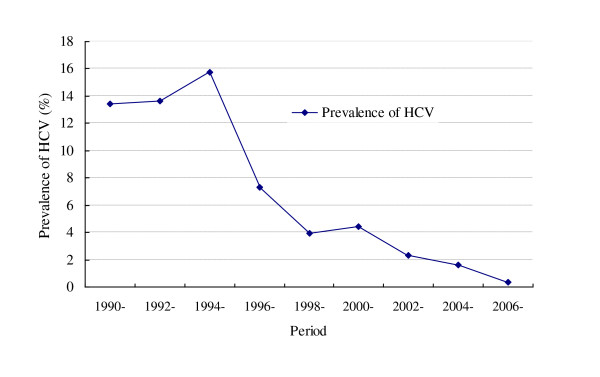
**Prevalence of HCV infection among blood donors at different study period**.

As presented in Table 2, 179 studies were divided into 9 groups according to the study period, and Figure 4 was drawn on the prevalence of HCV in each group. During 1994-1995, the prevalence rate reached the highest, which was 15.78% (95% CI: 12.21%-19.75%). Since 1995, the rates showed a decreasing trend among blood donors, even as low as 0.36% (95% CI: 0.09%-0.81%) during 2006-2010.

**Table 2:** Prevalence of HCV infection among blood donors at different study period

Study period	No. of studies	Prevalence of HCV % (95%CI)	Heterogeneity		Model
				
			*I^2^*	*P *value	
1990-1991	8	13.42(5.79, 23.62)	98.90%	0.00	REM
1992-1993	48	13.66(9.93,17.87)	99.70%	0.00	REM
1994-1995	57	15.78(12.21,19.75)	99.80%	0.00	REM
1996-1997	23	7.34(5.40,9.54)	99.30%	0.00	REM
1998-1999	13	3.97(2.67,5.54)	98.50%	0.00	REM
2000-2001	7	4.45(0.48,12.08)	99.90%	0.00	REM
2002-2003	14	2.34(1.17,3.87)	99.50%	0.00	REM
2004-2005	4	1.65(0.56,3.29)	97.40%	0.00	REM
2006-2010	5	0.36(0.09,0.81)	99.30%	0.00	REM

REM: random effect model.

#### Blood type

As displayed in Table 3, among blood type A, B, AB, and O donors, the HCV infection rates were 8.18% (95% CI: 4.55%-12.77%), 7.58% (95%CI: 4.26%-11.73%), 8.15% (95%CI: 4.64%-12.54%), and 7.85% (95%CI: 4.64%-11.82%), respectively. There was no significant statistical difference among four blood types (χ^2 ^= 4.97, *P *= 0.17).

**Table 3:** Prevalence of HCV infection among blood donors of different blood type

Blood type	No. of studies	Prevalence of HCV % (95%CI)	Heterogeneity		Model
				
			*I^2^*	*P *value	
A	14	8.18(4.55,12.77)	98.80%	0.00	REM
B	14	7.58(4.26,11.73)	98.80%	0.00	REM
AB	14	8.15(4.64,12.54)	94.90%	0.00	REM
O	14	7.85(4.64,11.82)	98.80%	0.00	REM

REM: random effect model.

#### Donation type

As seen in Table 4, HCV infection rate of voluntary blood donors and paid blood donors were 0.97% (95% CI: 0.79%-1.16%) and 15.53% (95% CI: 13.28%-17.91%) respectively. There was significant statistical difference (Z = 325.65, *P *< 0.01). The prevalence of HCV infection differed significantly (Z = 142.22, *P *< 0.01) among plasma donors and whole blood donors, which was 7.90% (95%CI: 6.44%-9.51%) and 33.95% (95%CI: 29.80%-38.17%), respectively.

**Table 4:** Prevalence of HCV infection among blood donors of different donation types

Donation type	No. of studies	Prevalence of HCV% (95%CI)	Heterogeneity		Model
				
			*I^2^*	*P *value	
voluntary donation	73	0.97(0.79,1.16)	99.60%	0.00	REM
paid donation	89	15.53(13.28,17.91)	99.70%	0.00	REM
whole blood donors	53	7.90(6.44,9.51)	98.50%	0.00	REM
plasma donors	64	33.95(29.80,38.17)	99.40%	0.00	REM

REM: random effect model.

## Discussion

As a blood-borne pathogen, HCV virus was frequently detected among paid blood donors in China in the early 1990s [9]. To improve the safety of blood supply and reduce the risk of transfusion-transmitted diseases, the Chinese government has outlawed the use of paid blood donors since 1998. As a result, Chinese blood banks now rely on various other methods to recruit blood donors, mostly on employer-organized donors and true voluntary donors [274]. This transition in the blood donor recruitment methods has been associated with a gradual decrease in the prevalence of anti-HCV among donors. In addition, an HCV RNA screening strategy was implemented in 2010 in all Chinese blood banks, which has also contributed to the decline of HCV prevalence rate. This review shows that the pooled prevalence of HCV infection among blood donors was 8.68% (95% CI: 8.01% to 9.39%) from 1990 to 2010, significantly higher than the estimated 3.2% in the general population of China [1]. It is noteworthy that before 1998 the pooled prevalence of HCV infection was 12.87% among blood donors, but dramatically decreased to 1.71% after 1998. In economically developed regions, the decreasing trend was more prominent due to effective control measures.

Our results showed significant geographic difference of the prevalence of anti-HCV. Compared with other regions, North and Central China had relatively higher anti-HCV positive rates among blood donors, accounting for 13.45% and 14.74%. Meanwhile, the lowest epidemic rate was found in South China with a percentage of 2.88%. Notably, during the period through 1990 and 1998, the prevalence rates were commonly at high level among different regions of China. Especially in Henan and Hebei, the rates even reached 35.04% and 29.26% respectively. After 1998, the general epidemic rates have rapidly decreased, benefited from the government's prohibition of using paid blood donors. However, the reduction of HCV prevalence was not that obvious in North and Central China. Possible reasons for this lack of reduction may include larger population migration, poor economic conditions, higher HCV infection rates in the general populations, or limited sampling.

According to the national epidemiological survey of viral hepatitis from 1992 to 1995, HCV infection rate among the general population increased gradually with age, but the prevalence rate had no significant difference between male and female [275], which is consistent with our findings that significant difference was found between two age groups but not between gender. The findings indicated that both male and female had the same susceptibility to HCV infection, while the older had increased chance of being HCV-infected. Since the paid donors were usually driven by economic benefits, the association of age with HCV infection may also be explained by increased exposure chances with a greater number of instances of blood donation times or longer duration of blood donation. It is early for a conclusive explanation until systematic investigations are performed and causal relation underneath is revealed [35,61,65,153,179].

By and large, the prevalence rate of HCV infection showed a rising trend among blood donors from 1990 to 1995, but significantly decreased from 1996 to 2010 (Figure 3). Our results revealed an outbreak of HCV infection in blood donors around 1995. As recalled, lots of plasma collection stations were established in different regions before 1995. However, the majority of them were illegal and severe cross-contamination on plasmapheresis frequently occurred due to those commonly existed nonstandard operations, such as neglecting sterilization, lacking accurate detection method of anti-HCV, improperly usage of non-disposable needles. In some places, the prevalence rate of HCV was reported as high as 80% [275]. Given the urgency of the situation, in 1995 the government implemented strict management on blood stations. Besides, the detection technology of anti-HCV got greatly improved with better sensitivity and specificity in the following years [276]. With the implementation of Blood Donation Law of China in 1998, real voluntary blood donors replaced paid donors and became a steady and major source of the blood supply. All these measures lead to the great achievements in control and prevention of HCV infection. Nowadays reports of HCV infection among the true voluntary blood donors could rarely been seen in China.

Our study showed that among blood type A, B, AB, and O donors, the prevalence of HCV infection were 8.18%, 7.58%, 8.15%, and 7.85%, respectively. No significant difference was found between blood types and the epidemic rate of HCV, indicating that blood type is not associated with susceptibility to HCV infection. This finding was consistent with the studies of Lu KQ [52], Zhou ZD [119], and Pu SF [269]. While it was a different story in the study of Ye C [239], in which type O blood donors were reported to have a higher infection rate, and type AB showed relatively lower rate. Uneven distribution with blood type was also seen in Rui ZL's study, in which type A blood donors were reported to have a higher rate [131]. However, the mechanism between blood type and HCV infection remained undefined, which may be related to red cell immune adherence function among persons with different blood types, but it need further study [131].

Numerous research has showed that paid blood donors are more likely to be infected with HCV than both employer-organized donors or true voluntary donors. Our results confirmed that HCV infection rate in paid blood donors was significantly higher than in voluntary blood donors (15.53% *vs *0.97%). Those paid donors who were attracted by high compensation and chose to donate blood in illegal blood stations, also risked a greater risk of cross-contamination. The prevalence rate among plasma donors was significantly higher than among whole blood donors (33.95% *vs *7.90%), possibly due to cross-contamination of blood collection equipment by HCV positive plasma donors [77]. The elimination of paid plasma and whole blood donation could contribute to a reduction in HCV infection among blood donors.

Several limitations in our study need to be addressed. First of all, the studies were observational and blood donors were not randomly chosen. Therefore selection bias and confounding seems inevitable. Secondly, many of our data were extracted from studies written in Chinese, which makes it difficult for non-Chinese reviewers, editors, and readers to trace back to the original materials. Thirdly, our ability to assess study quality was limited by the fact that many studies failed to offer detailed information of selected subjects or valid data on important factors. Besides, as with all meta-analyses, this study has potential limitation of publication bias. Negative trials are sometimes less likely to be published. However, we have confidence on our results since the included literatures were mostly from multi-resources and had large sample size, which should reduce publication bias to some extent.

## Conclusions

This meta-analysis provides a comprehensive and reliable data on the prevalence and trend of HCV infection among blood donors. The pooled epidemic rate of HCV infection has rapidly decreased after 1998, though some provinces still showed relatively high prevalence. Achievements and lessons in previous work indicated that long-term, comprehensive and effective interventions and preventions are urgently needed. In particular, implementing and enforcing the "Blood Donation Law" and promoting HCV screening, diagnosis, and treatment among blood donors are very important measures to control the transmission of HCV infection. In addition, the key to reduce the incidence of HCV infection among blood donors is to encourage true voluntary blood donation, pay more attention to exclude those high-risk persons from the volunteers, and eliminate cross-infection completely when collecting single plasma.

## Competing interests

The authors declare that they have no competing interests.

## Authors' contributions

XFG, QC, XS, JS and RBY were involved in the design, literature searching, assessment of study quality, and drafted the manuscript. JS and RBY revised critically the manuscript. XFG, QC and ZHP performed statistical analysis and critically revised the manuscript. KQD, NL and XC constructed the maps. LW and NW critically revised original study design and the manuscript. All the authors read and approved the final manuscript.

## Pre-publication history

The pre-publication history for this paper can be accessed here:

http://www.biomedcentral.com/1471-2334/11/88/prepub
